# Nano-Encapsulated Black Bean-Cultivated *Cordyceps militaris* Attenuates PM- and LPS-Induced Airway Inflammation

**DOI:** 10.3390/nu18132043

**Published:** 2026-06-23

**Authors:** Hyo-Min Kim, Hye-Jin Park

**Affiliations:** Department of Veterinary Medicine, College of Veterinary Medicine, Konkuk University, Seoul 05029, Republic of Korea; hyomin0401@konkuk.ac.kr

**Keywords:** *Cordyceps militaris*, *Rhynchosia nulubilis*, nanoencapsulation, particulate matter, anti-inflammatory mechanisms

## Abstract

Background/Objectives: Exposure to particulate matter (PM) containing bacterial endotoxins triggers inflammation and oxidative stress in the respiratory epithelium. In this study, we investigated chitosan nanoparticle-loaded *Cordyceps militaris* grown on germinated *Rhynchosia nulubilis* (GCN) as a potential functional food-derived ingredient against PM- and lipopolysaccharide (LPS)-induced cellular damage in human lung epithelial cells. Methods: This study employed an integrative approach combining GCN analysis with bioinformatics methods using a PM- and LPS-induced pulmonary cellular inflammation model. Gene Expression Omnibus (GEO) transcriptomic datasets and Cytoscape-based network analysis were utilized to identify key hub genes and signaling pathways associated with PM- and LPS-induced pulmonary inflammation, which were subsequently validated by RT-PCR and Western blotting. Results: Nano-encapsulation significantly improved the antioxidant capacity and storage stability of the extract compared with non-encapsulated *Cordyceps militaris* grown on germinated *Rhynchosia nulubilis* (GRC). GCN markedly attenuated PM- and LPS-induced cytotoxicity and intracellular reactive oxygen species (ROS) production in a dose-dependent manner, resulting in a therapeutic index approximately 4.5-fold higher than that of GRC under PM and LPS co-exposure. Bioinformatics analysis identified inflammation-related genes and pathways associated with PM- and LPS-induced pulmonary responses, primarily enriched in tumor necrosis factor (TNF)-related inflammatory pathways, Toll-like receptor signaling, and cytokine signaling. Consistent with these findings, GCN suppressed the expression of C-X-C motif chemokine ligand 2 (*CXCL-2*) and tumor necrosis factor-alpha (TNF-α) mRNA and inhibited mitogen-activated protein kinase (MAPK)-mediated activator protein-1 (AP-1) and nuclear factor-kappa B (NF-κB) signaling pathways in human type II alveolar epithelial cells (A549). Conclusions: Collectively, nano-encapsulation enhanced the stability and bioactivity of *Cordyceps militaris*-based extracts, suggesting that GCN may have potential as a functional food-derived candidate ingredient to protect airway epithelial cells against inflammation and oxidative stress induced by PM and LPS. As this study was conducted using an in vitro A549 epithelial cell model, further validation in physiologically relevant systems is needed to confirm its translational applicability.

## 1. Introduction

Air pollution is regarded as a major environmental health concern and is widely recognized as a risk factor associated with respiratory dysfunction [1]. Atmospheric particulate matter (PM) is categorized into coarse particles (2.5–10 μm), fine particles (<2.5 μm), and ultrafine particles (<100 nm), depending on particle size [2]. Among these, PM2.5 (fine PM characterized by an aerodynamic diameter of less than 2.5 μm) can deeply penetrate the respiratory tract and reach the alveolar region because of its small aerodynamic diameter. PM2.5 exposure has been shown to promote inflammation, oxidative stress, excessive generation of reactive oxygen species (ROS), and apoptotic cell death in lung tissues [1,3]. PM2.5 contains various toxic constituents, such as transition metals, volatile organic compounds (VOCs), polycyclic aromatic hydrocarbons (PAHs), carbonaceous particles, and endotoxins derived from Gram-negative bacteria [4]. These components contribute to airway epithelial dysfunction and persistent inflammatory responses associated with environmental exposure [1].

PM can carry bacterial endotoxins such as lipopolysaccharide (LPS), which are abundant in organic dust and polluted air. LPS adsorbed onto PM particles can intensify PM-induced pulmonary injury by triggering cytokine release, neutrophil recruitment, and epithelial damage, even at concentrations typical of ambient environments—effects that exceed those attributable to PM alone [1,5]. Of clinical relevance, individuals with pre-existing respiratory infections may face compounded risks upon PM and LPS co-exposure: while microbial pathogens, including *Haemophilus influenza*, *Moraxella catarrhalis*, *Streptococcus pneumonia*, *Staphylococcus aureus*, and *Pseudomonas aeruginosa* already induce airway epithelial injury and immune dysregulation, the additional burden of LPS-carrying PM is expected to further exacerbate the severity of infection-driven lung disease [1,6,7,8,9,10,11]. In support of this, our previous study demonstrated that unsterilized PM, which retains microbial components including LPS, exhibited significantly greater cytotoxicity in RAW264.7 macrophages compared with sterilized PM, suggesting that the bacterial components of PM, particularly LPS, play a critical role in amplifying PM-induced inflammatory responses [12]. This mechanistic rationale directly underpins the PM and LPS co-exposure model used in this study, which was designed to reflect the compounded pulmonary stress experienced under environmentally and clinically relevant conditions.

Corticosteroids, antihistamines, and adrenergic receptor agonists are commonly used for the symptomatic management of airway inflammation and respiratory symptoms associated with environmental exposure, including exacerbations of chronic obstructive pulmonary disease (COPD) and asthma [13,14]. However, these therapies are primarily intended for symptom control and may not sufficiently address persistent oxidative stress and inflammation induced by long-term PM exposure [15,16]. In addition, prolonged use of these medications may be associated with adverse effects, including immunosuppression, metabolic disturbances, cardiovascular complications, and reduced therapeutic responsiveness [15,17]. In this context, natural products and food-derived bioactive substances have gained increasing attention because of their favorable safety profiles, low toxicity, and potential for long-term dietary application. Therefore, the development of functional ingredients capable of modulating oxidative stress and inflammatory responses represents a promising approach for mitigating PM-associated adverse health effects [18,19].

*Cordyceps militaris* (*C. militaris*) is an edible medicinal mushroom traditionally consumed in Asia and valued for its beneficial health-related properties, especially in supporting respiratory function [20,21]. It contains a variety of bioactive compounds, including cordycepin, adenosine, polysaccharides, D-mannitol, ergosterol, and carotenoids [22,23,24,25,26]. Previous research has reported that these bioactive substances possess diverse pharmacological properties, including anti-inflammatory, antioxidative, immunomodulatory, and neuroprotective effects, highlighting the therapeutic potential of *C. militaris* as a functional food and medicinal material [27,28,29]. These bioactive substances reportedly exert both antioxidant and anti-inflammatory activities, contributing to the potential role of *C. militaris* as a functional ingredient for maintaining respiratory and cardiovascular health [22,23,24]. However, *C. militaris* grows slowly in lepidopteran larvae and requires complicated cultivation processes. Therefore, natural fruiting bodies are scarce, making them costly to collect and difficult to produce in large quantities. This limits their use as a functional food. Therefore, artificial cultivation of *C. militaris* has become a favorable alternative [30]. In this regard, germinated *Rhynchosia nulubilis* (*R. nulubilis*) has attracted attention because it possesses excellent nutritional characteristics, including various physiologically active compounds such as saponins, isoflavones, and anthocyanins, as well as a high protein content [31]. In particular, *R. nulubilis* exhibits anti-inflammatory effects [32], and its potent antioxidant properties may enhance immunity and help alleviate lung inflammation [33,34]. Therefore, we studied *C. militaris* cultured using germinated *R. nulubilis*, provided by CARI Co., Ltd. as an alternative to insect-based substrates. Extracts of *C. militaris* cultivated on germinated *R. nulubilis* (GRC) contain diverse bioactive constituents, including adenosine, polysaccharides, and cordycepin derived from *C. militaris*, together with phenolic constituents, including proanthocyanidins and isoflavones from germinated *R. nulubilis*. Our previous studies have demonstrated that cultivating *C. militaris* on germinated soybeans enhances the accumulation of major bioactive constituents, including adenosine, cordycepin, and β-glucan, and generates novel isoflavonoid derivatives such as glycitein 7-O-β-D-glucoside 4″-O-methylate (CGLM), genistein 7-O-β-D-glucoside 4″-O-methylate (CGNMI), genistein 4-O-β-D-glucoside 4″-O-methylate (CGNMII), and daidzein 7-O-β-D-glucoside 4″-O-methylate (CDGM). These extracts exhibit multiple biological activities, including anti-inflammatory, anti-apoptotic, immune-enhancing, and anti-allergic effects [33,35,36,37]. However, the oral application of GRC extract is restricted due to poor membrane permeability of its hydrophilic bioactive constituents, such as cordycepin, adenosine, anthocyanins, and β-glucan [38,39,40,41], the low aqueous solubility of genistein [42], chemical instability during digestion and absorption [43,44], and poor stability during storage [45,46].

Chitosan (CHI) nanoparticles represent a promising system for drug delivery to protect bioactive compounds because of their low toxicity, biocompatibility, biodegradability, controlled release properties, and enhanced storage stability [44,47,48]. In our previous studies, GRC was successfully encapsulated in CHI nanoparticles (GCN), as confirmed by systematic physicochemical characterization, including zeta potential analysis, encapsulation and loading efficiencies (EE/LE), and structural assessments using X-ray diffraction (XRD) and Fourier transform infrared spectroscopy (FT-IR). The optimized formulation exhibited a nanoscale particle diameter of approximately 146 nm and a positive zeta potential of up to +30.68 mV, indicating favorable colloidal stability and successful incorporation of GRC. Specifically, the optimized GCN formulation (8 mg/mL GRC) exhibited a mean particle diameter of 146.1 ± 54 nm, which is considered favorable for cellular uptake and bioavailability enhancement. In addition, the GCN formulation exhibited a positive zeta potential of +30.68 mV and encapsulation and loading efficiencies of 31.4 ± 1.35% and 7.6 ± 0.33%, respectively [12,49]. These physicochemical characteristics support the successful encapsulation of GRC within the chitosan nanoparticle system. In addition, enhanced cellular uptake and improved intestinal absorption were verified, further demonstrating the functional advantages of nanoparticle encapsulation [12,49]. Based on these findings, CHI nanoparticles are expected to prevent premature degradation of encapsulated compounds by gastric acid and digestive enzymes upon oral administration and to enhance the storage stability of polyphenols, anthocyanins, and genistein, which are sensitive to temperature and light. This protection helps preserve antioxidant activity and allows the major bioactive compounds of GRC to be absorbed more efficiently in the body, thereby improving biological activity and oral bioavailability [47,48,50]. 

Recent studies from our laboratory demonstrated that GCN exhibits enhanced cellular uptake, intestinal absorption, and protective effects against PM- or PM- and LPS-induced lung inflammatory responses under both in vitro and in vivo conditions [12,49]. However, the synergistic impact of PM and bacterial endotoxins, including LPS, on airway inflammatory responses and the molecular pathways underlying GCN-mediated protection under combined exposure conditions remains insufficiently understood. In this study, publicly available Gene Expression Omnibus (GEO) transcriptomic datasets and network-based bioinformatics analysis were utilized to identify key genes and signaling pathways related to PM- and LPS-induced pulmonary inflammation and to predict potential molecular targets of GCN [51,52]. These findings were experimentally validated in human type II alveolar epithelial cells (A549). Accordingly, this study was designed to: (i) identify major inflammatory genes and pathway markers associated with PM- and LPS-induced pulmonary inflammatory responses using a bioinformatics-based strategy; (ii) clarify the molecular mechanisms responsible for the protective effects of GCN under PM and LPS exposure conditions; and (iii) experimentally validate its anti-inflammatory efficacy in A549 cells.

## 2. Materials and Methods

### 2.1. Preparation of GRC Extract for GCN Synthesis

The hot-water GRC extract was provided by the Cell Activation Research Institute (CARI, Seoul, Republic of Korea). A total of 10 kg of GRC was extracted using 100 L of ionized water at 100 °C for 4 h, followed by filtration. The first and second extracts were combined and concentrated for use as the test sample. The concentrated extract was obtained at a yield of 17.8% (*w*/*w*), corresponding to 1.78 kg from the initial 10 kg of GRC. Furthermore, GRC was mixed with 80% methanol at a 1:20 (*w*/*v*) ratio and extracted using a microwave oven (700 W) for 2, 4, and 6 min, respectively. The extracts were filtered through a 0.45 μm membrane, and the obtained supernatants were used in subsequent analyses. GRC encapsulation with chitosan nanoparticles was carried out as described previously [12,49]. For nanoparticle synthesis, GRC extract was prepared at 8 mg/mL before encapsulation. The GCN solution was centrifuged at 22,250× *g* for 30 min (Avanti J-E; High-Speed Centrifuge; Beckman Coulter, Brea, CA, USA). The centrifuged pellet was then resuspended in 5 mL of 10% sucrose solution for lyophilization.

### 2.2. Total Antioxidant Capacity (TAC) Assay

The TAC of the sample was measured by the phosphomolybdenum method [36]. 100 μL of sample solution (100 mg/mL) was mixed with 400 μL of reagent solution containing 28 mM sodium phosphate (Sigma-Aldrich, St. Louis, MO, USA), 0.6 M sulfuric acid (Daejung Chemicals & Metals Co., Ltd., Siheung-si, Republic of Korea), and 4 mM ammonium molybdate (Samchun Pure Chemical Co., Ltd., Pyeongtaek, Republic of Korea). The mixture was incubated at 95 °C for 90 min and then cooled to room temperature. The absorbance of each sample was read at 695 nm using a microplate reader (Epoch, Biotek Instruments, Inc., Winooski, VT, USA). Ascorbic acid (Sigma-Aldrich, St. Louis, MO, USA) served as the reference standard, and ascorbic acid equivalents (AAE) were determined from a standard calibration curve. The total antioxidant activity of the samples was reported as μg of AAE per mg of sample (μg AAE/mg sample).

### 2.3. High-Performance Liquid Chromatography (HPLC) Analysis for Adenosine

Adenosine in GRC was quantified by HPLC (Agilent 1100 liquid chromatography system; Agilent Technologies, Santa Clara, CA, USA). A Zorbax ODS C18 column (250 mm × 4.6 mm i.d., 5 μm, Agilent Technologies, Santa Clara, CA, USA) was operated at room temperature. The mobile phase was composed of 15% methanol with 0.01 M KH_2_PO_4_ and served as the mobile phase with a flow rate of 1.0 mL/min. The injection volume was 10 μL, and UV detection was carried out at 254 nm. The retention time of adenosine was 11.7 min. Calibration curves were generated by injecting five to six concentrations of standard compounds and plotting peak area against analyte concentration. The calibration curve for adenosine was y = 26.983x − 21.426, with an r^2^ value of 0.9997 across a concentration range of 1–200 μg/mL.

### 2.4. PM2.5 Sample Preparation

PM collection and extraction were performed according to a previously published protocol [33]. PM2.5 particles were collected from the underground parking lot of Gachon University (Seongnam-si, Gyeonggi-do, Republic of Korea), a site selected to obtain PM reflective of vehicle exhaust-derived particulate pollution. Collection was performed twice per week over two independent two-month sampling periods: 26 April–26 June 2022, and 9 April–9 June 2023. Fine particles were collected using HEPA filters. Briefly, each filter was placed in a 50 mL tube containing 10 mL of 75% ethanol and sonicated for 30 min at 4 °C with an Ultrasonic Processor Sonicator (SONICS, Newtown, CT, USA). Subsequently, particles smaller than 2.5 μm were separated using Whatman filter paper (1005-055, Maidstone, Kent, UK). The resulting supernatant was concentrated under reduced pressure and stored at −80 °C until later use.

### 2.5. Cell Culture and Cell Viability Assay

A549 cells were obtained from the Korean Cell Line Bank (KCLB, Seoul, Republic of Korea). Cells were maintained in RPMI 1640 (Welgene, Seoul, Republic of Korea) containing 10% fetal bovine serum (Welgene) and 1% penicillin and streptomycin (Welgene). The cells were grown in 75 cm^2^ cell culture flasks at 37 °C under humidified conditions with 5% CO_2_. According to a previous study [53], cell viability was assessed using the Cell Counting Kit-8 (CCK-8) (Dojindo Laboratories, Kumamoto, Japan). A549 cells were seeded in a 96-well plate (1 × 10^4^ cells/well) and cultured overnight. After 1 h of pretreatment with either GCN (25, 50, 100, 200 μg/mL) or GRC (25, 50, 100, 200 μg/mL), cells were incubated with or without PM (50, 100, and 200 μg/mL) and LPS (5.5 ng/mL) for 24 h (Sigma Aldrich, St. Louis, MO, USA). The final concentration of DMSO (Sigma Aldrich, St. Louis, MO, USA), which was used as the solvent for PM, was kept below 0.05%, and the control group received an equivalent concentration of DMSO. Then, cells were treated with CCK-8 solution to assess cell viability by measuring absorbance at 450 nm (Epoch microplate reader; BioTek Instruments, Inc., Winooski, VT, USA). The 50% effective concentration (EC50) and 50% cytotoxic concentration (CC50) values were calculated using ED50plus v1.0 software (Mario H. Vargas, Instituto Nacional de Enfermedades Respiratorias, Mexico), which applies straight-line regression to cumulative concentration-response curves expressed in μg/mL. CC50 was determined from extract-only cell viability assays using GCN or GRC treatment alone, and represented the concentration associated with a 50% reduction in viable cells. In contrast, EC50 was determined under PM- and LPS-induced inflammatory conditions following GCN or GRC treatment, and represented the concentration required to achieve 50% of the maximal observed protective effect on cell viability. In addition, the therapeutic index (TI) was calculated according to the following Equation (1):

(1) $$TI=\frac{CC50}{EC50}$$

### 2.6. Dynamic Light-Scattering (DLS)

The particle size distribution of PM2.5 suspended in ultrapure water was determined by dynamic light scattering with a DynaPro Plate Reader II (Wyatt Technology, Santa Barbara, CA, USA). Before analysis, the suspension was sonicated at 20% amplitude for 40 s to disperse particle aggregates, after which DLS measurements were performed [54].

### 2.7. ROS Assay

Intracellular ROS production was evaluated using 2′,7′-dichlorodihydrofluorescein diacetate (DCFH-DA) as previously described [49]. This compound is oxidized by ROS and converted into the fluorescent derivative 2′,7′-dichlorodihydrofluorescein (DCF) according to the supplier’s instructions (ab113851; Abcam, Cambridge, UK). A549 cells were seeded at 3 × 10^5^ cells/well and cultivated overnight. The cells were pretreated with either GCN (200 μg/mL) or GRC (200 μg/mL) for 1 h. Cells were treated with PM (200 μg/mL) and LPS (5.5 ng/mL) for 4 h, followed by a single wash with 1× dilution buffer. H_2_O_2_ served as the positive control and was applied for 4 h. The final concentration of DMSO was kept below 0.05%, and control cells received the same concentration of DMSO. Afterward, they were incubated with 25 µM DCFH-DA for 45 min. DCF-DA fluorescence was detected using Nikon Eclipse Ti Fluorescence microscopy (Point Grey Research, Richmond, BC, Canada). To assess DCFH-DA fluorescence intensity, measurements were performed with a fluorescence plate reader (EnSpire, PerkinElmer, Waltham, MA, USA) at excitation and emission wavelengths of 485 and 535 nm, respectively [33].

### 2.8. Acquisition of Microarray Data and Identification of Differentially Expressed Genes (DEGs)

The Gene Expression Omnibus (GEO; http://www.ncbi.nlm.nih.gov/geo/, accessed on 30 September 2025) database contains publicly available functional genomics datasets, including gene expression profiles and microarray datasets. In this study, the GSE193958 (3 normal lung tissues and 2 cigarette smoke (CS)- and LPS-exposed lung tissues) and GSE41684 (normal lung tissues and CS- and LPS-exposed lung tissues) datasets were retrieved from the GPL6246 ([MoGene-1_0-st] Affymetrix Mouse Gene 1.0 ST Array) and GPL1261 ([Mouse430_2] Affymetrix Mouse Genome 430 2.0 Array) platforms. DEGs were identified, and data were normalized using GEO2R (http://www.ncbi.nlm.nih.gov/geo/geo2r/, accessed on 30 September 2025), which enables users to obtain DEGs across experimental conditions by comparing two or more sample groups based on the limma R package and GEO query. For data normalization, the “force normalization” option was applied to ensure that samples displayed the same value distribution. The cutoff criteria utilized for screening DEGs between the case and control groups were |log (fold change; FC)| > 1 and adjusted *p*-value < 0.05 [55].

### 2.9. Collection of PM- and LPS-Induced Lung Inflammation-Related Genes

Target genes associated with major PM- and LPS-induced lung inflammatory conditions were acquired from GeneCards as described by Yin et al. [56]. The GeneCards database (https://www.genecards.org/, accessed on 30 September 2025) contains extensive information on annotated and predicted human genes through the automated integration of gene-centered data from approximately 200 web sources. “Chronic obstructive pulmonary disease”, “asthma”, “acute respiratory distress syndrome”, and “pneumonia” were chosen as keywords to identify protein-coding genes with GeneCards Inferred Functionality Score (GIFtS) ≥ 30 [56,57]. GIFtS is a GeneCards-derived score that estimates gene functionality based on integrated annotations and accumulated biological evidence associated with individual genes. A threshold of GIFtS ≥ 30 was applied to exclude poorly characterized genes while retaining functionally annotated disease-associated targets for downstream analysis, consistent with thresholds used in previous respiratory disease-related network pharmacology studies [56,57,58].

### 2.10. Identification of Common and Hub Genes

We used Venny 2.1.0 (https://bioinfogp.cnb.csic.es/tools/venny/, accessed on 30 September 2025) to identify overlapping genes among the DEGs from GSE193958 and GSE41684, as well as target genes from GeneCards, thereby identifying genes related to PM- and LPS-induced lung inflammatory responses. The common genes obtained were entered into the online database Search Tool for the Retrieval of Interacting Genes (STRING v. 12.0; https://string-db.org/cgi/input.pl, accessed on 30 September 2025) to analyze significant relationships among them by constructing protein–protein interaction (PPI) networks using a confidence score cutoff value of 0.9, after which unconnected nodes were filtered out [59]. The common genes were then prioritized using the Maximal Clique Centrality (MCC) and Degree algorithms in the CytoHubba plugin of Cytoscape v. 3.10.4 (Cytoscape Consortium, San Diego, CA, USA) software for selection of the top 10 hub genes [60,61].

### 2.11. Gene Ontology (GO) and Kyoto Encyclopedia of Genes and Genomes (KEGG) Pathway Functional Analysis

Common genes were entered into the Database for Annotation, Visualization, and Integrated Discovery (DAVID v. 2023q4) for GO and KEGG pathway enrichment analyses. GO enrichment analysis was conducted for three categories: biological process (BP), cellular component (CC), and molecular function (MF). DAVID was used to visualize gene enrichment in the BP, CC, MF, and KEGG pathways, with a significance threshold of *p* < 0.05.

### 2.12. Reverse Transcription Polymerase Chain Reaction (RT-PCR)

Reverse transcription PCR was conducted as previously reported [62,63]. A549 cells were seeded in 6-well plates at a density of 5 × 10^5^ cells/well and cultured overnight. Cells were pretreated with either GCN or GRC (200 μg/mL) for 1 h and subsequently stimulated with PM (200 μg/mL) and LPS (5.5 ng/mL). The final concentration of DMSO was kept below 0.05%, and control cells received the same concentration of DMSO. Total RNA was isolated from A549 cells using TRIzol reagent (Invitrogen, Carlsbad, CA, USA). Reverse transcription was performed with a ReverTra Ace qPCR RT kit (Toyobo Biologics Inc., Osaka, Japan) according to the manufacturer’s instructions. PCR amplification consisted of an initial denaturation step at 94 °C for 2 min, followed by 35 cycles of denaturation at 94 °C for 30 s, annealing at 55 °C for 30 s, and extension at 68 °C for 1 min. The following primers were applied: human *TNF-α* forward 5′-AAG CCT GTA GCC CAT GTT GTA G-3′, reverse 5′-GAT GGC AGA GAG GAG GTT GAC-3′; human *CXCL-2* forward 5′-CTC CTT GCC AGC TCT CCT C-3′, reverse 5′-AGC TTT CTG CCC ATT CTT GAG-3′; human *TRPC6* forward 5′-AAT TGT GCA TAC CCT CCT GC-3′, reverse 5′-TGG CAG TTT GGA TGA GCT AC-3′; and human *GAPDH* forward 5′-GAG AAG GRG GGG CTC ATT T-3′ (R = A or G, according to IUPAC degenerate nucleotide nomenclature), reverse 5′-AGT GAT GGC ATG GAC TGT GG-3′ (Bionics, Seoul, Republic of Korea) (Bioneer, Daejeon, Republic of Korea). To ensure reliable quantification of gene expression changes, TNF-α, *CXCL-2*, and transient receptor potential canonical 6 (TRPC6) mRNA levels were normalized to the housekeeping gene glyceraldehyde-3-phosphate dehydrogenase (GAPDH), and the calculated values were expressed relative to those of the control group.

### 2.13. Western Blot Analysis

Western blot analysis was performed according to previously reported methods [63]. Briefly, cells were lysed using radioimmunoprecipitation assay buffer (Cell Signaling Technology, Danvers, MA, USA) and subsequently homogenized. Proteins were collected by centrifugation at 14,000× *g* for 10 min. Protein concentrations were determined using the Pierce bicinchoninic acid Protein Assay Kit (Thermo Fisher Scientific, Waltham, MA, USA). Equal amounts of protein were separated by 10% sodium dodecyl sulfate-polyacrylamide gel electrophoresis. The separated proteins were subsequently transferred onto nitrocellulose membranes (Bio-Rad Laboratories, Inc., Hercules, CA, USA) and blocked in 5% bovine serum albumin for 1 h at 25 °C. The membranes were then incubated overnight at 4 °C in Tris-buffered saline containing Tween (20 mM Tris, 500 mM sodium chloride, pH 7.6, 0.1% Tween 20) containing primary antibodies against phosphorylated (p)-IκB (1:1000; Cell Signaling), phospho-NF-κB (1:1000; Cell Signaling), and β-actin (1:1000; Cell Signaling) diluted according to the manufacturer’s protocol. The membranes were subsequently incubated for 1 h with a horseradish peroxidase-conjugated anti-rabbit IgG secondary antibody (1:2000; Cell Signaling). The blots were developed using an enhanced chemiluminescence detection solution (EzWestLumi plus, ATTO Corporation, Tokyo, Japan), and images were acquired and quantified with Odyssey LCI Image software version 4.0 (LI-COR Biosciences, Lincoln, NE, USA). The blots shown are representative of at least three independent replicates.

### 2.14. Statistical Analysis

All experiments were conducted with three independent biological replicates (*n* = 3), and data are expressed as mean ± standard deviation (SD). Prior to statistical analysis, data normality was evaluated using the Shapiro–Wilk test. Statistical differences among multiple groups were assessed by one-way analysis of variance (ANOVA), followed by Tukey’s honestly significant difference (HSD) post hoc test. For single pairwise comparisons between GRC and GCN groups, an independent samples *t*-test was used. Significance was set at *p* < 0.05, *p* < 0.01, and *p* < 0.001. All statistical analyses were conducted using SPSS version 12 (IBM, Chicago, IL, USA).

## 3. Results

### 3.1. Formation of Chitosan Nanoparticles from Different GRC Extracts and Evaluation of Antioxidant Potential and a Bioactive Compound

**Figure 1:**
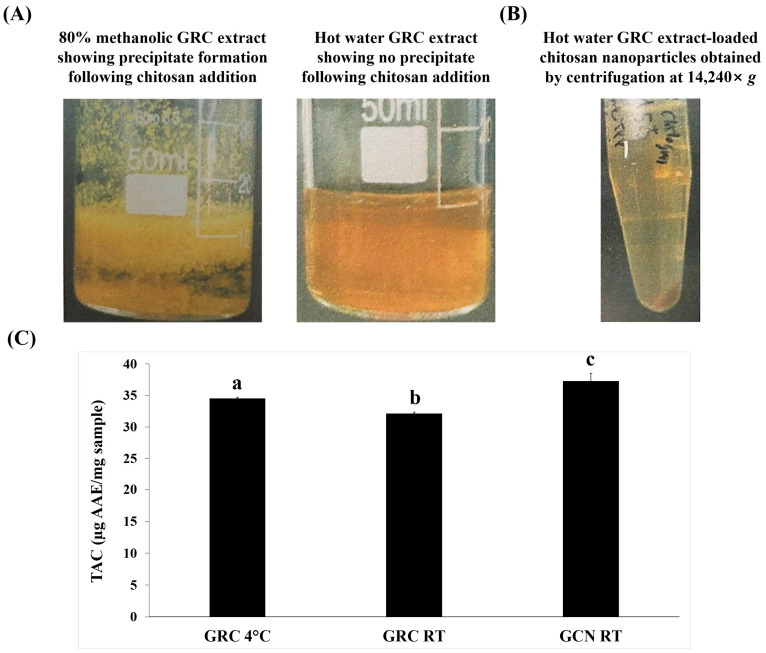
Preparation of *Cordyceps militaris* grown on germinated *Rhynchosia nulubilis* (GRC)-encapsulated chitosan nanoparticles and their antioxidant activity and storage stability. (**A**) Comparison of 80% methanolic GRC extract (**left**) and hot water GRC extract (**right**) following chitosan addition; precipitate formation was observed in the methanolic extract, whereas no precipitate was detected in the hot water extract. (**B**) Hot water GRC extract-loaded chitosan nanoparticles obtained by centrifugation at 14,240× *g*. (**C**) Total antioxidant capacity (TAC) of GRC stored at 4 °C and at room temperature, and chitosan nanoparticle-loaded-GRC (GCN) stored at room temperature for 20 days. TAC was expressed as μg of ascorbic acid equivalents (AAE) per mg of sample. The experiments were repeated three times and results are presented as mean ± standard deviation (SD). Statistically significant comparisons between groups were analyzed using one-way analysis of variance (ANOVA) with Tukey’s honestly significant difference (HSD) post-test. a–c, Bars with different letters differ significantly at *p* < 0.05 by Tukey HSD test.

During the chitosan nanoparticle preparation, precipitate formed with the 80% methanolic GRC extract, indicating unsuccessful nanoparticle formation (Figure 1A). No precipitate formed with the hot-water GRC extract, and centrifugation after tripolyphosphate (TPP) addition yielded a nanoparticle pellet, which was used for subsequent preparation (Figure 1B). To compare the antioxidant activity and storage stability of GRC and GCN, three different samples-GRC stored at 4 °C, GRC stored at room temperature, and GCN stored at room temperature-were analyzed using a TAC assay. The results demonstrated that GCN exhibited higher antioxidant capacity than GRC and showed improved stability after 20 days of storage at room temperature (Figure 1C). In addition, the adenosine content in GRC was 0.36 ± 0.00 mg/g (Table 1).

### 3.2. GCN Improves PM- and LPS-Induced Reduction in A549 Cell Viability

**Figure 2:**
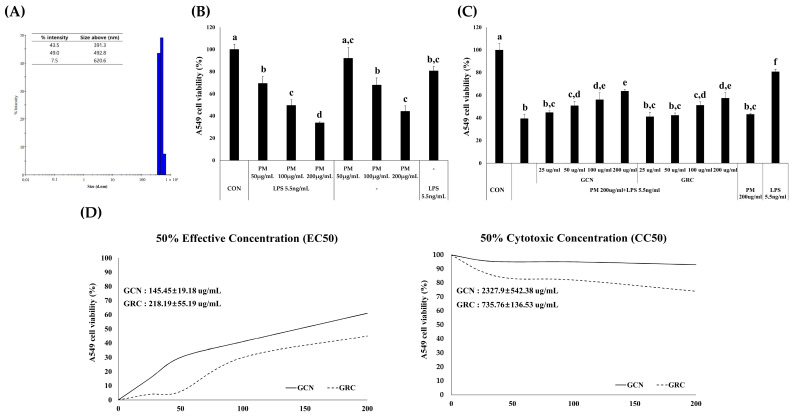
Effects of GRC, GCN, particulate matter (PM), and lipopolysaccharide (LPS) on the viability of type II alveolar epithelial cells (A549). (**A**) Representative dynamic light-scattering (DLS) analysis data of PM2.5 suspension. (**B**) The viability of cultured A549 cells treated with various concentrations (25, 50, 100, and 200 μg/mL) of PM and LPS (5.5 ng/mL) for 24 h. LPS was used as a positive control. (**C**) Effects of GCN and GRC on the viability of cultured A549 cells exposed to PM (200 μg/mL) and LPS (5.5 ng/mL). A549 cells were treated with various concentrations (25, 50, 100, and 200 μg/mL) of GCN and GRC for 24 h. PM and LPS were used as positive controls. A549 cell viability was assessed using Cell Counting Kit-8 (CCK-8) assays. (**D**) Dose–response curve of GCN and GRC on A549 cell viability. Data are expressed as mean ± SD from three independent biological replicates (*n* = 3). For (**B**,**C**), bars with different letters (a–f) differ significantly at *p* < 0.05 by Tukey HSD test following one-way ANOVA. Therapeutic index (TI) values derived from (**D**) were compared between the GRC and GCN groups using an independent samples *t*-test (*p* < 0.01).

DLS analysis showed that the PM2.5 particles ranged from 391.06 ± 0.18 nm to 492.50 ± 0.22 nm, with an average size of 318 ± 33.7 nm (*n* = 3; Figure 2A). Co-treatment with PM and LPS significantly decreased A549 cell viability in a concentration-dependent manner compared with the PM-treated groups (50, 100, and 200 μg/mL) and the untreated control (*p* < 0.05) (Figure 2B). GCN significantly restored cell viability in PM- and LPS-co-treated A549 cells and exhibited a greater protective effect than GRC at 200 μg/mL (*p* < 0.05) (Figure 2C). The EC50 indicates the concentration of GCN or GRC required to elicit a half-maximal protective effect against PM- and LPS-induced reduction in A549 cell viability. By contrast, CC50 indicates the concentration at which GCN or GRC alone reduces cell viability by 50%. The EC50 of GRC in A549 cells was 218.19 ± 55.19 μg/mL, whereas the EC50 of GCN was 145.45 ± 19.18 μg/mL, representing a reduction of approximately 1.5-fold for GCN. In addition, the CC50 values of both GRC and GCN in A549 cells were estimated to be >200 μg/mL under the tested experimental conditions (Figure 2D). No significant cytotoxicity was observed for GCN at concentrations of 25–200 µg/mL, and these concentrations were therefore selected for subsequent experiments (Supplementary Figure S1). The TI, which reflects the relative balance between cytotoxicity and protective efficacy [64,65], was used to compare the therapeutic margins of GCN and GRC. The TI was calculated to compare the relative safety of GCN and GRC, yielding a TI of 15.85 ± 1.67 for GCN and 3.49 ± 0.97 for GRC. The TI of GCN exceeds the commonly accepted minimum threshold of 10 for viable drug candidates, representing an approximately 4.5-fold increase compared with GRC, which indicates a statistically significant enhancement in safety and protective efficacy against PM- and LPS-induced reduction in A549 cell viability (independent samples *t*-test, *p* < 0.001), supporting the potential of GCN for further therapeutic development.

### 3.3. GCN Reduces PM- and LPS-Induced ROS Production in A549 Cells

**Figure 3:**
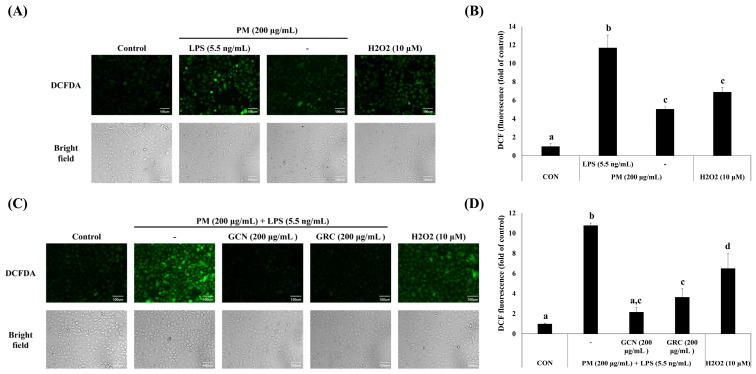
Scavenging effects of GCN on PM- and LPS-induced intracellular reactive oxygen species (ROS) in A549 cells. (**A**,**C**) Intracellular ROS were detected using fluorescence microscopy (Nikon Eclipse Ti microscope, Point Grey Research, Richmond, BC, Canada) after 2′,7′-dichlorodihydrofluorescein diacetate (DCF-DA) staining, using Metamorph software version 7.8 (Universal Imaging Corporation, West Chester, PA, USA; magnification = 200×; scale bar = 100 μm). Hydrogen peroxide (H_2_O_2_) was used as a positive control. (**B**,**D**) ROS scavenging effect of 200 μg/mL of GCN and 200 μg/mL of GRC on PM- and LPS-induced intracellular ROS in A549 cells. The intercellular level of ROS was measured using DCF-DA at a wavelength of 485/535 nm (Ex/Em). DCF fluorescence intensity was normalized to the control group and expressed as fold changes relative to the control. Data are presented as mean ± SD from three independent biological replicates (*n* = 3). Bars with different letters (a–d) differ significantly at *p* < 0.05 by Tukey HSD test following one-way ANOVA [12].

To establish a robust oxidative stress model, intracellular ROS production was first evaluated in A549 cells exposed to PM and LPS. Combined PM and LPS stimulation markedly increased intracellular ROS levels by 11.60 ± 0.54-fold compared with the control group and by 2.31 ± 0.16-fold compared with PM-treated cells alone (*p* < 0.05), demonstrating a synergistic effect of PM and LPS on oxidative stress induction (Figure 3A,B). Based on this validated oxidative stress condition, we next investigated the intracellular ROS scavenging activity of GCN. GCN treatment significantly suppressed PM- and LPS-induced intracellular ROS production, reducing ROS levels to 0.20 ± 0.05-fold relative to the stimulated group (*p* < 0.05), as indicated by decreased fluorescence intensity. Furthermore, GCN exhibited significantly greater antioxidant activity than GRC, with intracellular ROS levels in GCN-treated cells being 0.59 ± 0.00-fold lower than those observed in GRC-treated cells (*p* < 0.05; Figure 3C,D). Collectively, these results demonstrate that combined PM and LPS exposure effectively induces intracellular oxidative stress, and that GCN potently attenuates ROS overproduction under these oxidative stress conditions, supporting its antioxidant efficacy.

### 3.4. DEG Analysis Using GEO2R and DAVID for Biomarker Identification

**Figure 4:**
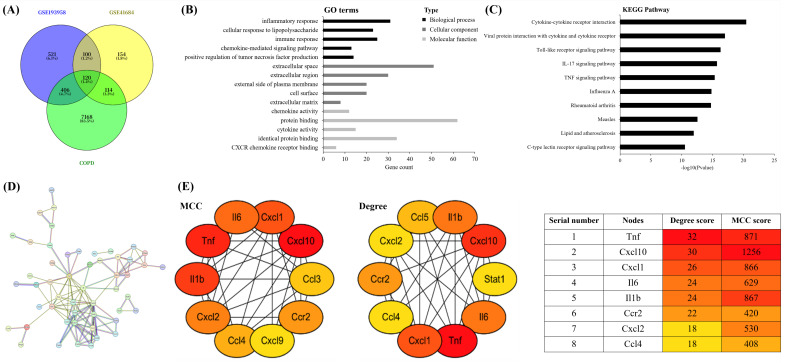
Functional enrichment analysis of common genes, followed by hub gene screening in the protein–protein interaction (PPI) network using CytoHubba plug-in. (**A**) Venn diagram showing the common genes between differentially expressed genes (DEGs) from GSE193958 and GSE41684 and chronic obstructive pulmonary disease (COPD)-related target genes. (**B**) Enrichment of biological process (BP), cellular components (CC), and molecular function (MF) of the common genes. (**C**) Kyoto Encyclopedia of Genes and Genomes (KEGG) pathway enrichment analysis of the common genes. (**D**) PPI network of the common genes was constructed using the STRING online database. Edges represent interactions between common genes. (**E**) Top 10 hub genes were ranked by CytoHubba using the following methods: Degree and Maximal clique centrality (MCC) scores.

We explored gene expression datasets to identify DEGs between PM- and LPS-treated and normal samples. Given the similarities in the chemical compositions of cigarette smoke and PM, both of which have been reported to induce overlapping oxidative stress and inflammatory responses in the respiratory system [66,67,68,69,70,71], the GSE193958 (*n* = 5) and GSE41684 (*n* = 8) datasets, which included CS- and LPS-treated samples, were examined, yielding 1300 (935 upregulated and 365 downregulated) and 651 DEGs (432 upregulated and 219 downregulated), respectively (|logFC| > 1, adjusted *p* < 0.05) [55]. Additionally, COPD-related target genes were retrieved from GeneCards (7808 protein-coding genes with GIFtS ≥ 30 to retain functionally annotated disease-associated genes) [56,57,58] and overlapped with these DEGs using a Venn diagram, identifying 120 common genes associated with major PM- and LPS-induced lung inflammatory conditions (Figure 4A). COPD was selected as a representative inflammation-related condition triggered by PM and LPS [4,72,73,74], including asthma, acute respiratory distress syndrome (ARDS), and pneumonia, because it showed significant overlap with the DEGs identified in the GSE193958 and GSE41684 datasets. To explore the key functional and biological characteristics of the 120 common genes, GO and KEGG pathway analyses were conducted using the DAVID database. The GO consists of three categories: BP, MF, and CC. The top five enriched terms in each category are shown in Figure 4B. In the BP analysis, common genes were significantly associated with inflammatory response (GO:0006954), cellular response to LPS (GO:0071222), immune response (GO:0006955), chemokine-mediated signaling pathway (GO:0070098), and positive regulation of tumor necrosis factor (TNF) production (GO:0032760). In MF analysis, genes were primarily enriched for chemokine activity (GO:0008009), protein binding (GO:0005515), cytokine activity (GO:0005125), identical protein binding (GO:0042802), and C-X-C motif chemokine receptor (CXCR) binding (GO:0045236). For the CC analysis, the genes were mainly involved in the extracellular space (GO:0005615), extracellular region (GO:0005576), external side of the plasma membrane (GO:0009897), cell surface (GO:0009986), and extracellular matrix (GO:0031012). KEGG pathway analysis revealed that the common genes were mainly associated with the cytokine-cytokine receptor interaction (mmu04060), toll-like receptor signaling pathway (mmu04620), TNF signaling pathway (mmu04668), IL-17 signaling pathway (mmu04657), and viral protein interaction with cytokine and cytokine receptor (mmu04061) (Figure 4C).

To investigate the PPIs among the 120 common genes, they were uploaded to the STRING database with the maximum number of interactors set to 0 and the minimum required interaction score set to the highest confidence level (≥0.9) [59]. This analysis generated a PPI network comprising 118 nodes and 1178 edges (Figure 4D). Next, Cytoscape plug-in CytoHubba was used to identify key hub genes within the network. The top 10 hub genes were identified by applying both the MCC and Degree algorithms to the PPI network, and the 8 overlapping genes were *TNF*, *CXCL-10*, *CXCL-1*, *IL-6*, *IL-1β*, *CCR2*, *CXCL-2*, and *CCL4*. These genes are likely to play critical roles in COPD induced by PM and LPS exposure (Figure 4E). Among them, *TNF*, *CXCL-10*, *IL-6*, *IL-1β*, and *CXCL-2* were also ranked among the top genes in the GEO2R differential expression analysis and are involved in the TNF signaling pathway (mmu04668).

### 3.5. GCN More Effectively Attenuates Cytokine and Chemokine Expression in PM- and LPS-Treated A549 Cells than GRC

**Figure 5:**
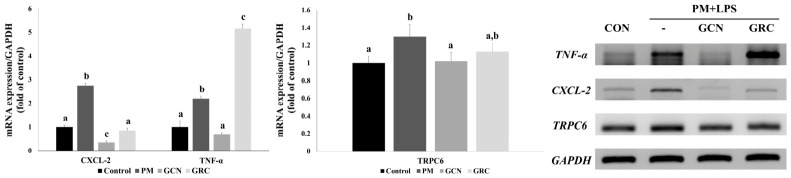
Effects of GCN on the levels of *CXCL-2*, *TNF-α*, and *TRPC6* mRNA expression in PM and LPS-stimulated A549 cells. A549 cells were pretreated with GCN (200 μg/mL) and GRC (200 μg/mL) for 1 h, followed by treatment with 200 μg/mL PM and 5.5 ng/mL LPS for 12 h. The levels of *CXCL-2*, *TNF-α*, and *TRPC6* mRNA were determined using Reverse Transcription Polymerase Chain Reaction (RT-PCR). Bar heights indicate the percentages of *CXCL-2*, *TNF-α*, and *TRPC6* mRNA at the indicated concentrations of samples, relative to the values determined in PM- and LPS-treated A549 cells. Data are presented as mean ± SD from three independent biological replicates (*n* = 3). Bars with different letters (a–c) differ significantly at *p* < 0.05 by Tukey HSD test following one-way ANOVA [33,53].

To evaluate the anti-inflammatory potential of GCN under inflammatory conditions, A549 cells were stimulated with PM and LPS. GCN significantly reduced the PM- and LPS-induced mRNA expression of the neutrophil chemoattractant *CXCL-2* by 87.9% ± 1.26 compared with the stimulated group (*p* < 0.05), demonstrating a strong inhibitory effect on chemokine induction. Although GRC also attenuated PM- and LPS-induced *CXCL-2* mRNA expression, GCN exerted a markedly greater inhibitory effect under the same conditions. In addition, GCN pretreatment significantly attenuated the mRNA expression of *TNF-α* in PM- and LPS-stimulated A549 cells (*p* < 0.05). Notably, *TNF-α* mRNA levels were reduced to 0.13 ± 0.00-fold relative to the PM- and LPS-treated group, indicating potent suppression of cytokine signaling by GCN (Figure 5). Given the established role of TRPC6 in *CXCL-2*-mediated neutrophil migration and chemotaxis, we further examined TRPC6 mRNA expression. Similarly, GCN significantly attenuated the PM- and LPS-induced upregulation of TRPC6 mRNA expression (*p* < 0.05), consistent with its inhibitory effect on *CXCL-2* mRNA expression.

### 3.6. GCN Exhibits Greater Inhibitory Effects than GRC on PM- and LPS-Induced Activation of Mitogen-Activated Protein Kinase (MAPK)-Mediated Nuclear Factor-Kappa B (NF-κB)/Activator Protein 1 (AP-1) Signaling Pathways

**Figure 6:**
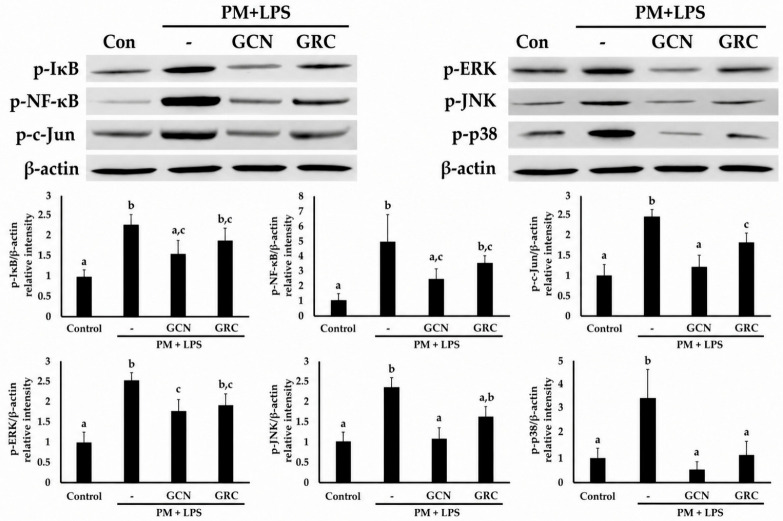
GCN inhibits PM- and LPS-induced inflammatory responses in A549 cells by suppressing the activation of NF-κB, AP-1, and MAPK pathways. A549 cells were pretreated with 200 μg/mL GCN or 200 μg/mL GRC for 1 h, followed by treatment with 200 μg/mL of PM and 5.5 ng/mL of LPS for 1 h. Whole-cell lysates were processed for Western blot analysis and probed with the indicated antibodies. p-IκB, p-NF-κB, p-c-Jun, p-ERK, and p-JNK protein expression levels in A549 cells were detected using Western blotting. Relative intensities are presented as mean ± SD from three independent biological replicates (*n* = 3). Bars with different letters (a–c) differ significantly at *p* < 0.05 by Tukey HSD test following one-way ANOVA.

Based on KEGG pathway analysis identifying the Toll-like receptor and TNF signaling pathways as key regulators of the NF-κB, MAPK, and AP-1 signaling cascades, this study was conducted to elucidate the molecular mechanisms underlying the anti-inflammatory activity of GCN. GCN significantly attenuated the PM- and LPS-induced phosphorylation of extracellular signal-regulated kinase (ERK), c-Jun N-terminal kinase (JNK), and p38 mitogen-activated protein kinase (p38 MAPK), as well as activation of the NF-κB signaling cascade, including phosphorylated inhibitor of κB (p-IκB), phosphorylated NF-κB (p-NF-κB), and the AP-1 signaling cascade, including phosphorylated c-Jun (p-c-Jun) in A549 cells (*p* < 0.05; Figure 6). Although both GCN and GRC (200 μg/mL) effectively reduced PM- and LPS-induced inflammatory signaling, GCN exerted significantly greater inhibitory effects than GRC on the activation of the MAPK (ERK, JNK, and p38 MAPK) and NF-κB signaling pathways, demonstrating its superior anti-inflammatory efficacy under inflammatory conditions (*p* < 0.05). Collectively, these findings indicate that GCN exerts anti-inflammatory effects in PM- and LPS-stimulated A549 cells via suppression of MAPK-mediated NF-κB and AP-1 signaling, supporting its potential as a functional food-derived ingredient for the prevention or alleviation of PM- and LPS-induced lung inflammation. The original, uncropped Western blot images can be found in Supplementary Figure S2.

## 4. Discussion

GRC has been reported to exert antioxidant, anti-inflammatory, and anti-apoptotic activities [12,35,36,49]. These activities are likely associated with the broad range of bioactive constituents contained in GRC. Specifically, GRC contains antioxidants, such as cordycepin, adenosine, and polysaccharides derived from *C. militaris*, as well as novel isoflavones, including CGNMII, CDGM, CGNMI, and CGLM, proteins, proanthocyanidins, and polyphenols derived from *R. nulubilis.* These *C. militaris*-derived bioactive compounds attenuate inflammatory responses and ROS production while enhancing antioxidant enzyme activity under PM exposure conditions, thereby reducing oxidative stress [24,26,33,35,36,75,76].

However, its biological effectiveness following oral intake may be limited by inadequate physicochemical stability and bioavailability, as key constituents such as phenolic compounds and cordycepin are susceptible to rapid metabolism and limited absorption in the gastrointestinal tract [38,43,77]. To address these limitations, we formulated GRC into a CHI-based nanoparticle to improve stability, protect labile bioactives from degradation, and enhance cellular uptake [12,49]. In the present study, both the hot-water extract of GRC and GCN exhibited substantial TAC, indicating that intrinsic GRC constituents substantially contribute to antioxidant potential. Notably, however, GCN retained significantly higher TAC than GRC after 20 days of storage, supporting the notion that chitosan-based encapsulation effectively stabilizes labile antioxidant components and preserves functional activity over time. The antioxidant capacity of GRC likely reflects the presence of water-soluble bioactives such as cordycepin [35,36,78], adenosine [78], polysaccharides [35,36], phenolic compounds [33,37,53,78], isoflavones, and anthocyanins, as previously characterized in our studies.

Importantly, the improved TAC retention observed for GCN suggests that nanoencapsulation protects these constituents from chemical and enzymatic degradation, thereby contributing to the improved stability of antioxidant activity. Given that excessive intracellular ROS generation is a critical upstream event in PM- and LPS-induced lung inflammation, the capacity of GCN to regulate cellular oxidative stress at the cellular level was subsequently evaluated using ROS-sensitive assays, which are described in the following sections. DLS analysis confirmed that the PM used in this study was less than 2.5 μm in size (Figure 2A), which is sufficiently small to penetrate the lower respiratory tract and reach the alveolar region [54]. Upon deposition in the alveoli, PM2.5 can directly interact with alveolar epithelial cells and exert its biological effects through a variety of organic and inorganic constituents [79]. PM2.5 contains organic components (e.g., PAHs, VOCs) and inorganic substances (e.g., sulfates, nitrates, and transition metals) that together promote ROS generation [80,81,82]. PAHs activate the aryl hydrocarbon receptor (AhR), inducing cytochrome P450 1A1 (CYP1A1), which generates superoxide and electrophilic quinones during PAH metabolism [83,84,85,86,87,88]. Quinones undergo redox reactions, resulting in superoxide anion (O_2_^•−^) and hydroperoxyl radicals (HOO•) formation [86,87,88]. Water-soluble ions such as sulfate (SO_4_^2−^) and nitrate (NO_3_^−^) present in PM2.5 indirectly enhance ROS production by promoting Fenton reactions. After inhaled PM reaches the alveoli, SO_4_^2−^ and NO_3_^−^ dissolve easily in the wet alveolar wall [89]. Particularly, SO_4_^2−^ binds to Fe^2+^, stabilizing it and enhancing the Fenton reaction, thereby increasing HO· production and inducing damage to alveolar cells [89,90,91]. Transition and heavy metals (e.g., Fe, Cu, Mn, Pb, As, Cd) further amplify oxidative stress and deplete antioxidant defenses [79,92,93,94,95,96,97]. Additionally, LPS in PM activates Toll-like receptor 4 (TLR4) signaling, upregulating NADPH oxidase 2 (NOX2) and NADPH oxidase 4 (NOX4), further increasing ROS generation in alveolar epithelial cells [98,99]. Accordingly, we observed that co-exposure to PM and LPS induced higher intracellular ROS levels in A549 cells than PM exposure alone, consistent with reports describing synergistic oxidative stress under combined exposure [100,101]. In addition, PM samples collected from the same location on different dates produced reproducible cytotoxic and inflammatory responses, supporting the consistency of the prepared PM samples.

Notably, GCN markedly reduced PM- and LPS-induced intracellular ROS levels compared with GRC, indicating that nanoencapsulation enhances the antioxidant efficacy of GRC in vitro. This enhanced activity may stem from improved cellular delivery and protection of labile compounds during uptake, thereby increasing the effective intracellular availability of antioxidant constituents [38,41,102]. Mechanistically, several GRC-derived constituents, such as cordycepin, adenosine, polysaccharides from *C. militaris*, and proanthocyanidins and polyphenols from *R. nulubilis,* as well as novel isoflavones (CGNMII, CDGM, CGNMI, and CGLM) from GRC, may contribute to ROS mitigation, enhancement of antioxidant enzyme activity, and suppression of inflammation [24,26,33,35,36,75]. Cordycepin as well as adenosine, the major bioactive nucleosides present in *C. militaris* [23,28], increase cellular antioxidant defenses by upregulating catalase (CAT), glutathione peroxidase, and superoxide dismutase (SOD), while diminishing production of O_2_^•−^ and hydrogen peroxide (H_2_O_2_), thereby protecting cells from oxidative damage [103,104]. Consistent with these reports, HPLC analysis confirmed the presence of adenosine in GRC, which may contribute to the observed antioxidant activity by enhancing cellular antioxidant defenses. Furthermore, polyphenols and polysaccharides, including β-glucan, enhance antioxidant defenses by increasing the activities of antioxidant enzymes such as CAT, SOD, and glutathione S-transferases (GSTs), and exhibit notable antioxidant activity by scavenging various ROS and chelating metal ions [105,106,107,108,109,110]. Collectively, these effects provide plausible biochemical support for the ROS-suppressive phenotype observed with GCN.

The reduction in intracellular ROS levels was accompanied by improved cell viability in PM- and LPS-exposed A549 cells, supporting a protective role against co-exposure-induced cytotoxicity. This finding is biologically meaningful because alveolar type II (AT2) cells play a crucial role in maintaining alveolar homeostasis, regulating surfactant production, and supporting epithelial regeneration. Excessive damage to AT2 cells may impair tissue repair processes and disrupt airway epithelial integrity. PM and LPS exposure have been reported to induce apoptotic cell death and functional impairment in AT2 cells, thereby aggravating oxidative stress and inflammatory responses in the lung [74,111,112,113,114,115,116]. Consistent with these reports, we observed a significant viability decline following PM and LPS co-treatment. Importantly, the higher TI of GCN compared with GRC indicates greater protective activity at lower cytotoxic concentrations, which aligns with improved uptake and bioavailability commonly associated with chitosan-based nanoparticles [12,49,117,118,119]. These results suggest that nanoencapsulation may have enhanced the cytoprotective effects of GRC and support the potential of GCN as a superior formulation for mitigating PM- and LPS-induced epithelial injury.

To further elucidate the inflammatory pathways associated with PM- and LPS-induced epithelial injury, transcriptomic analyses from two publicly available datasets (GSE193958 and GSE41684) derived from CS- and LPS-exposed lung tissues were integrated with inflammation-related gene profiles to identify key signaling pathways and regulatory factors involved in exposure-associated pulmonary inflammation. It should be noted that, due to the absence of publicly available PM- and LPS-specific transcriptomic datasets, CS- and LPS-based datasets were employed as a surrogate, given their chemically complex constituents and overlapping toxicological properties with PM [120,121,122]. Although the AKR subgroup in GSE193958 contained only two biological replicates per condition, this dataset was not used in isolation. To strengthen the reliability of candidate gene selection, DEGs from GSE193958 were integrated with those from the independent GSE41684 dataset, in which the 12 h CS- and LPS-treated C57BL/6 mouse group (*n* = 4) was selected based on its closest DEG profile similarity to the AKR subgroup in GSE193958. Accordingly, the combined CS and LPS exposure group comprised a total of six biological replicates (*n* = 6; *n* = 2 from GSE193958 and *n* = 4 from GSE41684), and overlapping DEGs identified across both datasets were used for downstream bioinformatic analysis, thereby improving the statistical robustness and reproducibility of the candidate gene selection. Although this represents an inherent limitation of the bioinformatics approach adopted in the present study, the candidate biomarkers and signaling pathways identified through this analysis were subsequently validated experimentally in a direct PM- and LPS-stimulated A549 cell model, supporting the translational relevance of the predicted molecular targets. Network analysis identified 120 genes enriched in cytokine–cytokine receptor interaction, Toll-like receptor (TLR) signaling, IL-17 signaling, and TNF signaling. These pathways converge on central inflammatory regulators, including the TLR4/myeloid differentiation primary response 88 (MyD88) axis and downstream MAPK, AP-1 and NF-κB cascades, which are known to mediate pro-inflammatory cytokine production under oxidative and inflammatory stress conditions [4,123,124,125,126,127,128]. Using CytoHubba, hub genes including TNF, *CXCL-10*, *IL-6*, *IL-1β*, and *CXCL-2*, which were consistently highly ranked and upregulated across datasets, were identified, suggesting key roles in inflammatory amplification and immune cell recruitment during exposure-associated airway inflammation [129,130,131,132]. The relevance of these hub genes and major inflammatory signaling molecules was experimentally validated in PM- and LPS-treated A549 cells using RT-PCR and Western blot analysis.

At the signaling level, PM- and LPS-induced oxidative stress and TLR4 activation can trigger MAPK (p38/JNK/ERK) and transcription factor cascades (NF-κB and AP-1), culminating in excessive cytokine/chemokine production, including TNF-α, *IL-1β*, *IL-6*, and *CXCL-2* [82,99,133,134,135]. This mechanism is consistent with our KEGG pathway analysis, which identified Toll-like receptor and TNF signaling as major inflammation-related pathways converging on the MAPK/NF-κB and AP-1 signaling networks (Figure 4C). This convergence reflects shared promoter architecture among these hub genes: the TNF-α promoter contains cooperative NF-κB (−510 bp) and AP-1 (−100 bp) binding sites, and p38 MAPK further stabilizes TNF-α mRNA through AU-rich element (ARE)-binding proteins (e.g., TTP/HuR); the *CXCL-2* promoter similarly harbors juxtaposed NF-κB (−80 bp) and AP-1 (−72 bp) elements, with JNK-mediated c-Jun phosphorylation at Ser63/Ser73 constituting an obligate activation step for *CXCL-2* transcription under LPS and oxidative stress conditions [136,137,138,139,140,141,142,143,144]. *CXCL-2* binds to C-X-C motif chemokine receptor 2 (CXCR2), promoting neutrophil migration and activation. Thus, *CXCL-2* is a key chemokine involved in neutrophil recruitment during pulmonary inflammation [129,130,131,132,145,146,147]. Inflammatory stimuli, including PM and LPS, increase CXCR2 expression and *CXCL-2* secretion in the lungs, and these responses are closely associated with TRPC6-mediated Ca^2+^ signaling, which promotes intracellular Ca^2+^ influx and neutrophil recruitment to the lung [148]. Consistent with our previous study, which proposed TRPC6 as a diagnostic biomarker for PM-induced COPD and reported elevated *TRPC6* mRNA expression in PM-exposed RAW 264.7 cells [149], we detected increased TRPC6 mRNA expression in alveolar epithelial cells exposed to PM and LPS.

Our results showed that GCN reduced the mRNA expression of TNF-α, TRPC6, and *CXCL-2* and decreased the phosphorylation levels of MAPK, NF-κB, and c-Jun in PM- and LPS-exposed lung epithelial cells. The suppression of p38, JNK, NF-κB/p65, and c-Jun phosphorylation observed in GCN-treated cells therefore provides a direct mechanistic explanation for the coordinated downregulation of these hub effector genes, as both TNF-α and *CXCL-2* are obligate transcriptional outputs of the NF-κB/AP-1 nodes suppressed by GCN.

In support of this mechanism, Reaxys-based target mining identified several GCN-derived bioactive compounds and their direct molecular targets associated with the MAPK/NF-κB/AP-1 signaling network. GCN contains several bioactive compounds, including cordycepin, β-glucan, isoflavonoids (e.g., genistein), and anthocyanins, many of which have been reported to possess anti-inflammatory properties [36,62,78,150,151,152,153]. Specifically, genistein was linked to the G-protein-coupled estrogen receptor (GPER), through which suppression of JNK/p38/ERK phosphorylation and NF-κB activation has been reported to reduce TNF-α mRNA expression [154]. Anthocyanins were associated with interleukin-17 receptor A (IL-17RA), where blockade of IL-17A/IL-17RA signaling attenuates p38 (MAPK14) phosphorylation [155]. In addition, adenosine was linked to adenosine receptor A2A (ADORA2A), activation of which suppresses *IL-1β*-induced ERK1/2 (MAPK3/MAPK1) phosphorylation and downstream inflammatory responses [156]. Collectively, these findings support a multi-target mechanism through which GCN modulates key inflammatory signaling pathways.

Cordycepin has been reported to inhibit the activation of NF-κB by suppressing IκB-α degradation and NF-κB nuclear translocation and to modulate ERK/JNK phosphorylation and LPS-TLR4 interactions [157,158]. Accordingly, cordycepin may affect two important regulatory points in *CXCL-2* transcription, NF-κB through IκB-α stabilization and AP-1 through JNK/c-Jun suppression, which may help explain the downregulation of *CXCL-2*, a hub gene identified by our network pharmacology analysis as a terminal effector associated with the TLR4-mediated MAPK/NF-κB/AP-1 signaling pathway suppressed by GCN. Previous studies have further demonstrated that cordycepin suppresses TLR4-dependent signaling and inhibits MAPK/NF-κB pathway activation, thereby reducing TNF-α and *CXCL-2* production [157,159]. β-glucan has been reported to attenuate LPS-driven inflammation by downregulating TLR4/MyD88 and suppressing ERK/JNK phosphorylation and NF-κB activation [160,161,162,163].

Since *CXCL-2* transcription depends on JNK-mediated c-Jun activation, the inhibitory effects of β-glucan on TLR4/MyD88 and JNK/AP-1 signaling may partly explain the reduced *CXCL-2* and TNF-α mRNA expression observed after GCN treatment in the present study [164,165,166,167]. Isoflavones such as genistein and daidzein may interfere with LPS-induced TLR4/MyD88 signaling and attenuate NF-κB activation, thereby reducing cytokine and chemokine production [168,169]. In addition, genistein and daidzein have been reported to interfere with TLR-4-associated signaling, leading to suppression of p38/JNK activation and downstream AP-1 signaling. These effects provide a plausible mechanistic basis for the reduced TNF-α and *CXCL-2* mRNA expression observed in GCN-treated cells [170,171,172,173]. Similarly, anthocyanins and related polyphenols attenuate intracellular ROS, a proximal activator of ASK1/TRAF6-mediated p38/JNK signaling [174], thereby suppressing downstream MAPK-dependent NF-κB and AP-1 signaling at an upstream oxidative stress checkpoint [174,175,176]. This reduction in ROS-dependent NF-κB/AP-1 activation may, in turn, contribute to the decreased transcription of *CXCL-2* and *CXCL-10*, both of which are co-regulated by NF-κB and AP-1 at the promoter level. Consistent with this interpretation, anthocyanins have also been reported to inhibit IL-17RA-associated p38 activation, providing an additional pathway through which MAPK-dependent inflammatory responses may be attenuated [155,177].

Although A549 monocultures are useful for evaluating epithelial inflammatory responses to PM and PM + LPS, they cannot reproduce interactions between pulmonary epithelial cells and macrophages or other immune cells. This limitation is particularly relevant under PM + LPS conditions, in which LPS-mediated innate immune activation may enhance PM-induced inflammation. Moreover, because the present findings have not been directly confirmed in vivo, they do not account for particle deposition and clearance, immune-cell recruitment, and tissue injury within the lung. Despite these limitations, as well as those associated with the acute exposure model, the use of a CS- and LPS-based transcriptomic dataset rather than PM-specific profiling, and the absence of more complex pollutant-combination models such as PM/heavy metals or PM/PAHs, this study provides the first demonstration that GCN more effectively attenuates PM- and LPS-induced inflammatory responses in human airway epithelial cells compared with GRC. Notably, nano-encapsulation not only enhanced the stability and bioavailability of the extract but also significantly improved its anti-inflammatory efficacy, as evidenced by the suppression of TNF-α and *CXCL-2* expression and the inhibition of MAPK-mediated NF-κB and AP-1 signaling pathways. These findings are further supported by bioinformatics-based identification of PM- and LPS-associated inflammatory gene networks, providing mechanistic insight into the molecular targets of GCN. Collectively, the present findings suggest that nano-encapsulation represents a promising strategy for enhancing the functional efficacy of *C. militaris*-based ingredients, and that GCN holds potential as a functional food-derived candidate for the prevention or alleviation of PM- and LPS-induced lung inflammation, warranting further validation in physiologically relevant models including primary airway epithelial cells’ and co-culture systems.

To address the limitations of the monoculture model, future studies will employ A549 co-culture systems incorporating RAW264.7 cells, THP-1-derived macrophages, and HL-60-derived neutrophil-like cells. RAW264.7 cells will be used to assess macrophage-epithelial interactions associated with inflammatory cytokine production and NF-κB activation, based on our previous observation of PM + LPS-induced inflammation in this cell line [12,178,179,180]. THP-1-derived macrophages will provide a human macrophage-like model for evaluating macrophage-mediated amplification of inflammation under PM + LPS conditions, relevant to innate immune responses associated with COPD exacerbation [181,182,183,184]. HL-60-derived neutrophil-like cells will be used to assess IL-8 production, migration, and neutrophil-associated inflammatory responses following PM and LPS stimulation [183,185]. These co-culture models will allow identification of immune cell types that respond more strongly to PM + LPS than to PM alone, and will determine whether the anti-inflammatory effects of GCN and GRC are maintained under co-culture conditions.

In this regard, future in vivo animal studies using PM- and LPS-induced murine lung injury models are planned to confirm the anti-inflammatory and antioxidant efficacy of GCN under physiologically relevant conditions. The immune cell populations showing greater sensitivity to PM + LPS in the co-culture experiments will be examined to determine whether they are also selectively increased or activated in lung tissue in vivo. The effects of GCN and GRC on these PM + LPS-responsive cell populations will be evaluated to assess whether the anti-inflammatory effects observed in vitro are reproduced in vivo. In addition, macrophage and neutrophil infiltration and lung tissue injury will be compared between PM-only and PM + LPS exposure groups, using F4/80 immunohistochemistry and H&E staining together with additional macrophage and neutrophil markers including F4/80 or CD68 and Ly6G or CD11b [12]. These analyses will determine whether GCN and GRC suppress PM + LPS-induced inflammatory cell infiltration and lung tissue injury.

In conjunction with these efficacy studies, targeted pharmacokinetic (PK) profiling will be conducted focusing on the tissue distribution of major bioactive constituents of GCN, including cordycepin, adenosine, and isoflavone-derived compounds, across multiple biological matrices including plasma, lung tissue, and bronchoalveolar lavage (BAL) fluid. While a complete absorption distribution metabolism excretion (ADME) characterization—encompassing metabolite identification and mass balance studies—would provide the most comprehensive mechanistic insight, such analyses are constrained in the current stage by the complexity of multi-component natural product matrices and the requirement for validated reference standards for each metabolite; accordingly, the initial PK focus will be placed on pulmonary distribution as the most clinically relevant parameter for this target indication. This multi-matrix PK approach will enable direct quantification of bioactive compound exposure at the target site, with the lung-to-plasma partition coefficient (K_p_) and BAL fluid drug concentrations serving as key indices of pulmonary bioavailability and target organ selectivity—parameters that cannot be inferred from plasma data alone [186,187,188,189]. Separately, oral bioavailability (F%) will be formally validated by comparing the area under the concentration-time curve (AUC) following oral administration with that following intravenous (IV) administration, providing direct evidence for the absorption-enhancing effect of chitosan nanoparticle encapsulation relative to the non-encapsulated GRC extract [187,190,191]. Collectively, these future studies will provide essential preclinical data to support the rational development of GCN as a therapeutic or nutraceutical intervention for pulmonary inflammatory diseases.

## 5. Conclusions

Overall, this study demonstrates that nano-encapsulation of GRC extract in chitosan nanoparticles enhances its stability and bioactivity, and provides mechanistic evidence for its protective activity against PM- and LPS-induced oxidative stress and inflammation in A549 cells. GCN pretreatment attenuated inflammatory signaling by regulating TLR4-mediated MAPK-NF-κB/AP-1 pathways and suppressing key hub genes, including TNF-α and *CXCL-2*. These findings suggest that GCN holds potential as a functional food-derived ingredient for respiratory protection against environmentally induced pulmonary inflammation, with TNF-α and *CXCL-2* identified as relevant molecular targets. Further validation across diverse cell lines and in vivo dietary intervention models will be needed to confirm its broader protective effects and translational applicability as a functional food component.

## Figures and Tables

**Table 1:** Adenosine content in GRC determined by HPLC.

Compounds	Concentrations of Analytes in 40 mg/mL GRC (μg/mL) ^a^	Contents (mg/g-GRC) ^b^
Adenosine	14.19 ± 0.07	0.36 ± 0.00

The mean values of three independent determinations are presented. ^a^ Values (μg/mL) indicate the concentration of analytes in a 50% methanol extract of GRC prepared at 40 mg/mL. ^b^ Contents (mg/g) in GRC on a dry weight basis.

## Data Availability

Dataset available on request from the authors.

## Supplementary Materials

The following supporting information can be downloaded at: https://www.mdpi.com/article/10.3390/nu18132043/s1, Figure S1: Assessment of GCN and GRC cytotoxicity in A549 cells; Figure S2: Uncropped Western blot images shown in Figure 6.
