# Supplementary material for: Nano-Encapsulated Black Bean-Cultivated Cordyceps militaris Attenuates PM- and LPS-Induced Airway Inflammation
Source: Nutrients. 2026 Jun 23;18(13):2043. doi: 10.3390/nu18132043 (PMC13363539; doi:10.3390/nu18132043)
Supplement: Supplementary file 1 [file nutrients-18-02043-s001.zip › nutrients-4297302-supplementary.pdf]

# Supplementary Materials

# Nano-Encapsulated Black Bean-Cultivated *Cordyceps militaris* Attenuates PM- and LPS-Induced Airway Inflammation

Hyo-Min Kim, Hye-Jin Park *

Department of Veterinary Medicine, College of Veterinary Medicine, Konkuk University, Seoul 05029, Republic of Korea

* Correspondence: nimpi79@hanmail.net

## Supplementary Figure S1

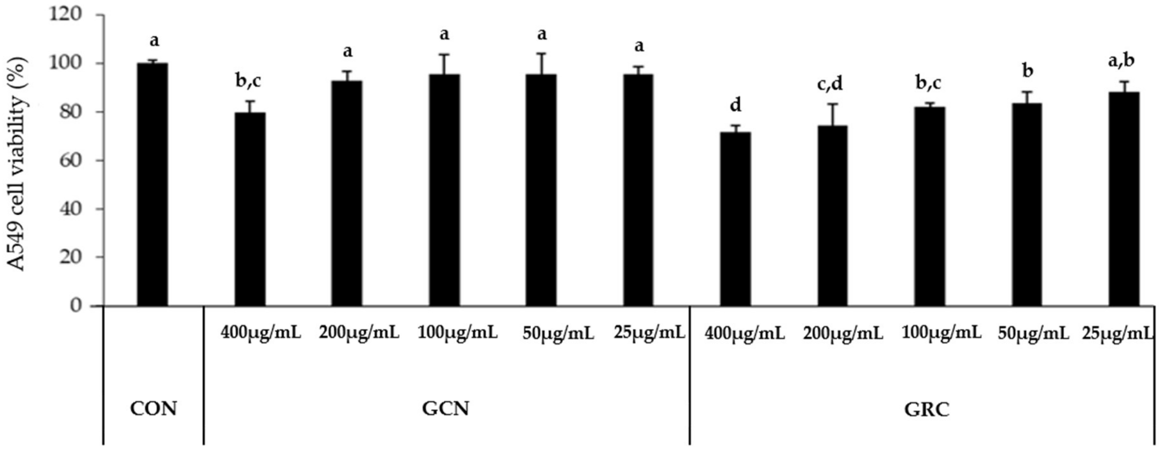


**Figure S1. Assessment of GCN and GRC cytotoxicity in A549 cells.** A549 cells were treated with GRC or GCN at concentrations of 25, 50, 100, and 200 μg/mL for 24 h. Cell viability was assessed using the Cell Counting Kit-8 assay. Data are presented as mean ± SD from three independent biological replicates (n = 3). Bars with different letters (a-d) differ significantly at $p < 0.05$ by Tukey HSD test following one-way ANOVA

## Supplementary Figure S2

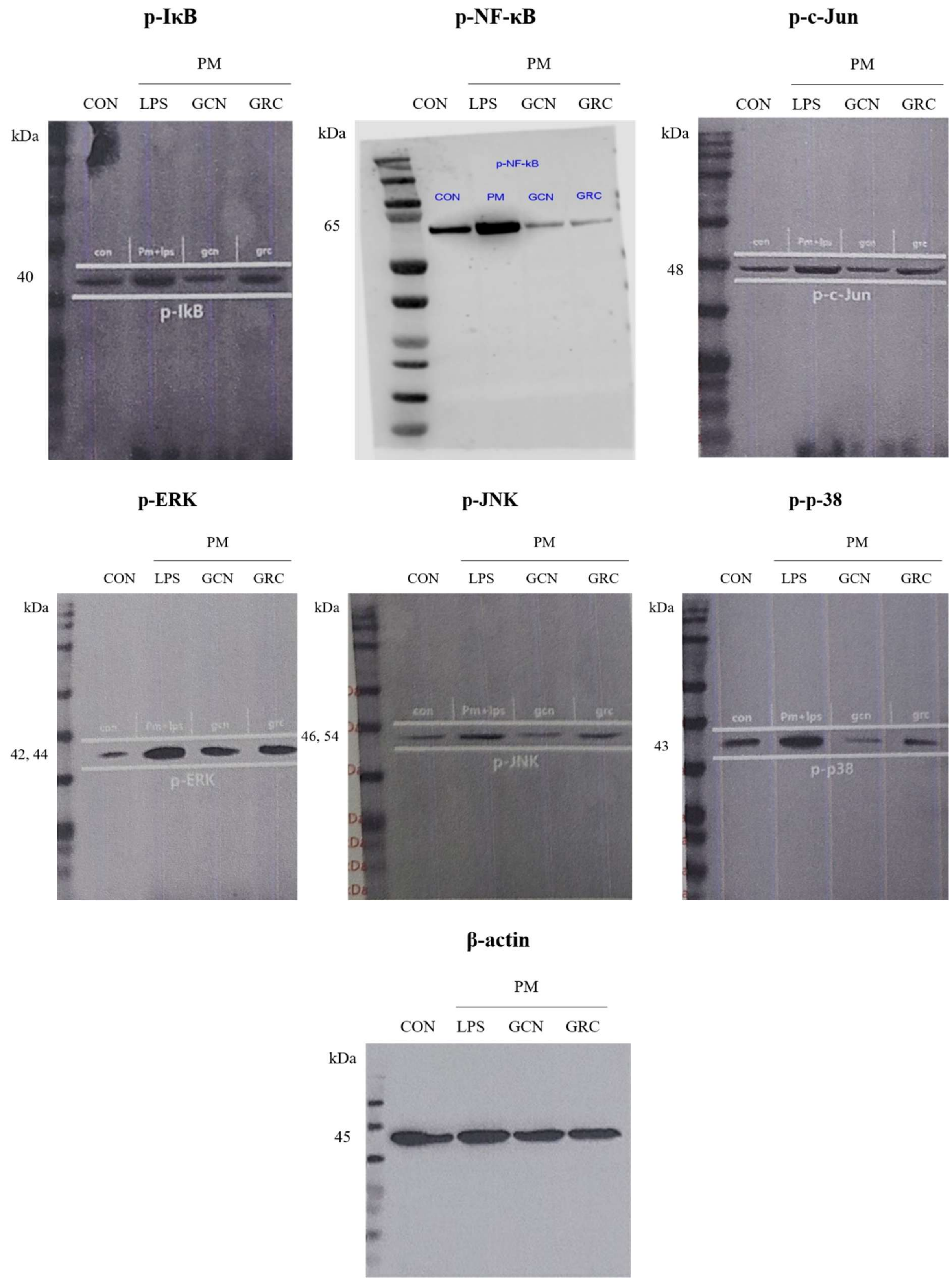


**Figure S2.** Uncropped Western blot images shown in Figure 6
